# Effects of Ramadan Fasting on Sleep and Physical Fitness among Young Female Handball Players

**DOI:** 10.3390/children11080954

**Published:** 2024-08-07

**Authors:** Mohamed Alaeddine Guembri, Ghazi Racil, Mohamed Tounsi, Chirine Aouichaoui, Luca Russo, Gian Mario Migliaccio, Yassine Trabelsi, Nizar Souissi, Johnny Padulo

**Affiliations:** 1 Research Unit: Physical Activity, Sport and Health, UR18JS01, National Observatory of Sport, Tunis 1003, Tunisia; alaaguembri@yahoo.fr; 2 Research Unit (LR 23JS01) “Sport Performance, Health & Society”, Higher Institute of Sport and Physical Education of Ksar Saîd, University of Manouba, Tunis 2010, Tunisia; ghazi_racil@yahoo.fr; 3 Research Laboratory: Exercise Physiology and Physiopathology: From Integrated to Molecular “Biology, Medicine and Health” (LR19ES09), Faculty of Medicine of Sousse, University of Sousse, Sousse 4000, Tunisia; m.tounsi@hotmail.fr (M.T.); chirineaouichaoui@yahoo.com (C.A.); trabelsiyassine@yahoo.f (Y.T.); niz.souissi@gmail.com (N.S.); 4 eCampus University, 22060 Novedrate, Italy; luca.russo2@uniecampus.it; 5 Department of Human Sciences and Promotion of the Quality of Life, San Raffaele Rome Open University, 00166 Rome, Italy; gianmario.migliaccio@uniroma5.it; 6 Maxima Performa, Athlete Physiology, Psychology and Nutrition Unit, 20126 Milan, Italy; 7 Department of Biomedical Sciences for Health, Università degli Studi di Milano, 20133 Milan, Italy

**Keywords:** Ramadan, fasting, adolescents, physical performance, sleep

## Abstract

Objectives: This study examines the potential impact of Ramadan fasting on sleep patterns and physical fitness in under 14 (U14) and under 17 (U17) female handball players. Methods: For this study, sixty-five female handball players’ (U14, *n* = 33 and U17, *n* = 32) sleep habits were assessed before (BR) and during the first (R1) and last weeks (R4) of Ramadan using: the Pittsburgh Sleep Quality Index (PSQI), the Insomnia Severity Index (ISI), and the Epworth Sleepiness Scale (ESS). Physical ability tests including balance and jumps were conducted throughout these periods.; Results: During Ramadan, both groups noted significant increases in PSQI and ISI scores during R1 (*p* < 0.01 and *p* < 0.05, respectively) and R4 (*p* < 0.05 and *p* < 0.05, respectively) compared to before Ramadan (BR). For the U14 group, a significant increase occurred in ESS scores during R1 (*p* < 0.01) and R4 (*p* < 0.05), whereas for the U17 group, this increase was limited over the R1 period (*p* < 0.05). In contrast, for all physical ability parameters, both groups showed no significant difference during R1 and R4; Conclusions: During adolescence, Ramadan fasting may alter sleep patterns in young female athletes but appears to have no effect on their performance in short-duration exercises.

## 1. Introduction

During the Islamic month of Ramadan, healthy Muslims are called upon to abstain from eating and drinking from dawn to sunset as part of their religious duty. This religious practice considers puberty as the age at which a child must begin fasting. In general, this intermittent fasting leads to changes in daily lifestyle, including eating habits, which can result in varying energy intake and metabolic and anthropometric changes [1,2,3]. These changes may also include alterations in sleep patterns, resulting in decreased nightly sleep duration [4,5] and shifts in circadian rhythms [6,7]. Additionally, studies have shown that Ramadan fasting can impact various aspects of human health, such as glucose metabolism, lipid profiles, and hydration status [8]. It can also influence cognitive functions, mood, and physical performance, depending on the duration and intensity of fasting [9]. Understanding these effects is crucial, especially for athletes who must balance the demands of their sport with the physiological challenges posed by fasting.

In the Tunisian context, it has been observed that most athletes, including adolescents, continue to fast while maintaining a school schedule and a weekly training frequency almost identical to those of the period before Ramadan [10]. In consequence, an increase in sleep disorders such as insomnia and sleepiness may appear, especially under their obligations of school requirements that force them to wake up too early, putting them in debt regarding sleep during the school week [11]. When this fact is repeated during the Ramadan period, it affects their physical performance [12,13], especially in the adolescent phase where sleep represents a primary factor for physical growth and maturation [14,15]. According to Van Helder et al. [16], the crucial importance of sleep was demonstrated in muscle recovery and regeneration, as well as in the enhancement of athletic skills, including anaerobic performance. The latter study established a significant link between sleep quality and anaerobic capacity, highlighting the importance of high-quality sleep in maintaining optimal sports performance. The underlying mechanisms of this impact may be associated with hormonal regulation, muscle recovery, and neuromodulation, among other factors.

To our knowledge, there is no established consensus on how Ramadan fasting affects the sleep patterns of physically active adolescent girls. Our study addresses this gap by focusing on this under-researched population. The value of our research is enhanced by examining potential variations in the anthropometric characteristics and sleep behaviors of adolescent girls, particularly those in different age groups.

In our previous research conducted under normal conditions [17], we observed significant differences in physical measurements between the U14 and U17 groups. This distinction underscored how the biological specificity related to age and sex influences sleep behaviors. Recognizing this, we were motivated to further investigate these effects during Ramadan, a period known for its unique fasting-related changes in daily routine and nutrition. Thus, our study aims to examine the impact of Ramadan fasting on certain sleep measures (PSQI, ISI, and ESS) among young female handball players aged under 14 (U14) and under 17 (U17).

In fact, handball requires several physical qualities such as balance and dynamic power. The development of these qualities from an early age is essential to the development of biological processes related to physical fitness and maturation [18]. Some studies have reported that, during Ramadan, intermittent fasting can exert a negative influence on physical performance, which is affected by different factors such as metabolic functioning [2], glucose regulation [7], circadian rhythm change [6,7], and dehydration problems [19]. Other studies, in contrast, have reported that Ramadan fasting did not impair muscle performance, especially following anaerobic exercises [12,13,20].

The above discrepancies could be attributed to factors such as the specific time of day when the tests were conducted or the duration of sleep deprivation [21]. In addition, these fluctuations may be due to the influence of psychological factors such as beliefs about maintaining usual training loads and mood, which may lead to early fatigue [12,22,23]. Another crucial aspect pertains to the frequency, intensity, and nature of the training undertaken during this month, demanding enhanced adherence and motivation [24,25]. All of the above factors have been associated with studies focusing on adult subjects whose lifestyles were different from those of youth. To our knowledge, few studies have specifically investigated the physical and behavioral states of young female handball players during Ramadan. The lack of research in this area is particularly notable given the unique challenges posed by fasting on young athletes, including potential impacts on sleep patterns, nutritional intake, and physical performance. We therefore hypothesize that physical performance could be altered during the month of Ramadan, especially during the fourth week, among female players under the ages of 14 (U14) and 17 (U17). Additionally, we hypothesize that fasting during Ramadan will affect both sleep habits and nutritional intake, which may consequently influence athletic performance.

## 2. Materials and Methods

### 2.1. Participants

**Figure 1:**
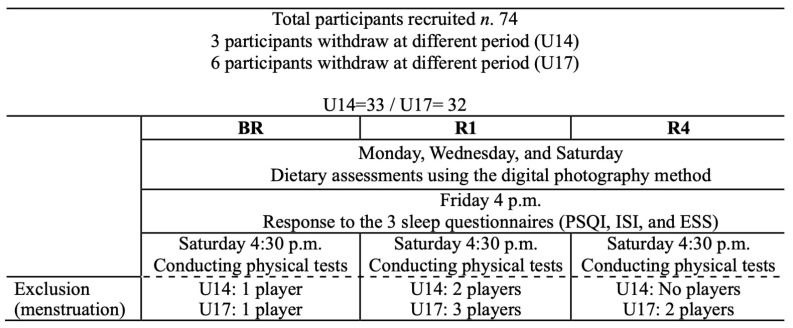
Study design for U14 and U17 female players during BR, R1, and R4.

Prior to commencing the intervention, all participants and their parents signed an informed consent form in accordance with the international ethical standards, in particular, the Declaration of Helsinki [26]. A total of 74 female handball players were initially recruited for this study. The participants were informed about the nature of the experiment, and those who volunteered were included in the study. The players were randomly divided into two groups based on their ages (U14: *n* = 33, 13.39 ± 0.4 years, stature 1.64 ± 0.02 m, body mass 56.98 ± 4.90 kg, BMI 21.19 ± 1.42 kg·m^−2^; U17: *n* = 32, 15.90 ± 0.69 years, stature 1.67 ± 0.04 m, body mass 59.55 ± 5.81 kg, BMI 21.71 ± 1.53 kg·m^−2^). To ensure consistent and representative data collection, participants were questioned about their menstrual status on the day of the tests, and this was performed by two women from the field of pediatrics. Those who were menstruating or suffering from illnesses or injuries were excluded. It should be noted that nine participants withdrew before the end of the study for personal reasons (three from the U14 group and six from the U17 group), and their data were not included in the statistical analysis for the different periods (Figure 1). The players are part of a local women’s handball association and undergo regular training of 6 h per week spread over four days, both before and during Ramadan. In this research, a power analysis was conducted prior to data collection using GPower software (Version 3.1.9.6) to determine the minimum sample size needed to ensure sufficient power to detect an effect [27]. We specified a desired power level of 80%, with a 5% α error and an effect size of 0.3, which are commonly accepted parameters in research studies. The output from G*Power indicated that a sample size of 65 female handball players was adequate for this study.

### 2.2. Anthropometric Measurements

Anthropometric measurements were taken by a physician. Body height was measured in centimeters without shoes, with heels together, and with the subject’s back parallel to the stadiometer (Model 214 height rod; Seca, Hamburg, Germany). Body mass (BM) was evaluated to the nearest 0.1 kg using a digital scale (Tanita, Tokyo, Japan), and body mass index (BMI = Mass [kg]/(Height [m])^2^) was determined. Each participant underwent bioelectrical impedance analysis (BIA) (Tanita Body Composition Analyzer Mode TBF-300, Tokyo, Japan) to measure their body fat mass (BF), lean mass (LM), and body mass index (BMI).

### 2.3. Dietary Measures

With the help of a specialized nutritionist, an attempt was made to monitor the dietary intake of all participants through an estimation of the portions consumed, especially the meal at the break of fasting and the Suhoor meal at the beginning of fasting. The assessment of food consumption was carried out using a validated digital photography method, as described by [28]. At the beginning of the study, uniform-sized plates and glasses were distributed to all participants to facilitate the quantification of food and liquid intake. Participants were asked to take photos of all foods and drinks consumed over a 24 h period (midnight to midnight) for three consecutive days, at three different times: before Ramadan (BR), during the first week of Ramadan (R1), and during the last week of Ramadan (R4). These images and detailed information were then analyzed by a nutritionist using Bilnut 4 software (SCDA Nutrisoft, Cerelles, France), with food consumption quantities referenced against data published in 1978 by the Tunisian Institute of Statistics.

The targeted nutritional parameters included:

Caloric Intake: Total daily caloric intake was measured using a 3-day dietary recall method, ensuring an accurate representation of the players’ typical consumption.

Macronutrient Distribution: The intake of carbohydrates, proteins, sugar, and lipids was analyzed to ensure balanced macronutrient distribution aligned with athletic performance needs.

### 2.4. Study Design

The experiment was conducted over 3 periods: 3 months before Ramadan (BR), during the first week of Ramadan (R1), and during the last week of Ramadan (R4). During the month of Ramadan (April 2021), the duration of fasting was approximately 15 h during R1 and 16 h during R4. On Friday, the fifth day of the BR, R1, and R4 weeks, all participants were asked to complete sleep questionnaires, describing their usual sleep patterns for the previous week, excluding the period when they were menstruating. Two examiners, each responsible for a group, were tasked with assisting in completing the questionnaires by explaining the relevant items.

On Saturday, all female players (U14 and U17) were required to undergo physical fitness tests starting at 4:30 p.m. for BR, R1, and R4, respectively. On the day of the experiment, they were interviewed about their menstrual cycle to find out if their test results may be biased. Those who were menstruating or in a bad mood were excluded. Three examiners were assigned to lead each workshop for a specific test. The first one oversaw the balance test, the second one the squat jump tests, and the last one the standing long jump test (SBJ) and the five jump test (FJT), noting that only these latter tests were conducted outside the handball hall, specifically on the long jump track.

Before starting, a general warm-up of 10 to 15 min was performed, consisting of short strides, joint mobilizations of the lower and upper limbs, and stretching [29]. A phase of familiarization with each event for all the participants was carried out.

### 2.5. Tests/Instruments/Measurements

#### 2.5.1. Sleep Questionnaires

The Pittsburgh Sleep Quality Index (PSQI) is a subjective sleep quality assessment questionnaire [30]. It consists of 19 self-evaluation items, grouped into 7 components, reflecting the following parameters: C1. Subjective sleep quality, C2. Sleep latency, C3. Sleep duration, C4. Usual sleep efficiency, C5. Sleep disturbance, C6. Use of sleep medication, and C7. Daytime dysfunction. Each component gives a score from 0 to 3. Specific values are as follows: a score of 0 indicates no problems, while a score of 2 or 3 indicates more significant issues. The addition of the 7 components gives us an overall PSQI score ranging from 0 to 21 points. A higher score indicates more significant sleep issues. A PSQI score ≥ 5 is an indicator of poor sleep quality.

The Insomnia Severity Index (ISI) is a self-reported subjective measure of the symptoms of insomnia and the level of anxiety caused by sleep disorders [31]. It is composed of seven items measuring the severity of insomnia: difficulty falling asleep, difficulty staying asleep, difficulty waking up too early, the degree of satisfaction with current sleep, the perception of daily dysfunction, and the degree of worry about sleep difficulties estimated by oneself. Each item is rated on a 5-point Likert scale ranging from 0 to 4, with answer options representing different levels of severity: 0 = Not at all, 1 = A little, 2 = Somewhat, 3 = Much, 4 = Very much. The addition of the scores of these 7 items provides us with a global score varying between 0 and 28, allowing us to classify the subjects according to the severity of insomnia: 0–7 = No insomnia, 8–14 = Subclinical or light insomnia, 15–21 = Moderate insomnia, and 22–28 = Severe insomnia.

The Epworth Sleepiness Scale (ESS) is a self-administered questionnaire containing 8 situations, with each situation composed of 3 scales from 0 to 3 evaluating the probability of dozing off [32]. The total score varies between 0 and 24, where a score exceeding 8 (ESS > 8) indicates excessive daytime sleepiness.

#### 2.5.2. Physical Fitness Tests

**The balance test (Open Eyes (OEB)/Closed Eyes (CEB))** was performed as described by [33]. The player was required to stand on a single leg of her choice, barefoot on a flat surface. The other leg was raised so that the foot of that leg was stuck next to the knee of the supporting leg. The player had to put her hands on her hips. The clock did not start until the player lifted the heel of their supporting leg. The player must maintain this position as long as possible while avoiding any kind of imbalance: lowering the heel of the supporting leg, turning the body in any direction, raising the hands from the hips, or hopping. When one of these actions was performed, the timer was stopped. Each player had 3 attempts to practice balance depending on the condition, either with open eyes (OEB) or closed (CEB). The test–retest reliability scores for OEB and CEB measurements were 0.82 and 0.77, with a respective 95% confidence interval of 0.48–0.84 and 0.69–0.85.

**Lower extremity explosive strength measurement:**

**The squat jump (SJ)** measured the players’ jump height from a starting position with knees flexed at 90° and hands on the hips. Using an Optojump (Microgate, Bolzano, Italy) system [34], the height performance was recorded in centimeters. The test–retest reliability and 95% confidence interval were 0.88 and 0.79–0.91, respectively.

**The five-jump test (FJT)** was conducted as described by [17]. Three attempts were made, and the best attempt was recorded. The test–retest reliability and 95% confidence interval were 0.91 and 0.81–0.95, respectively.

**The standing broad jump (SBJ)** was performed as described by [17]. Three attempts were made by each player, and the best distance was recorded. The test–retest reliability and 95% confidence interval were 0.85 and 0.78–0.89, respectively.

### 2.6. Statistical Analyses

Data analysis was performed using IBM SPSS software (version 26, IBM^®^, Armonk, NY, USA). The results were presented as the mean ± standard deviation (SD). The Kolmogorov–Smirnov method was used to check the normality of the data distribution. A repeated-measures analysis of variance (ANOVA) was used for all sleep (PSQI, ISI, and ESS), dietary, and physical fitness variables to compare the different periods (BR, R1, and R4) in U14 and U17 players separately. The effect size (ES) was also calculated (eta squared, η2) to aid the interpretation of the results (values of 0.01, 0.06, and above 0.14 were considered small, medium, and large, respectively). Additionally, Pearson’s bivariate correlation coefficient was used to investigate the relationship between sleep parameters and dietary parameters. Alpha values less than 0.05 were used as significance thresholds.

## 3. Results

After the collection of results, none of the groups showed significant changes from pre- to post-testing in all the anthropometric data (Table 1). During R1 and R4, there was a significant increase in both the PSQI score (*p* < 0.01 and *p* < 0.05, respectively) and the ISI score (*p* < 0.05, respectively) for U14 and U17 female players (Table 2). This increase coincides with a significant increase in energy, carbohydrate, and sugar intake (Table 3). Similarly, the ESS score significantly increased during R1 and R4 for U14 (*p* < 0.05, respectively) and only during R1 for U17 (*p* < 0.05) (Table 2). The different physical condition parameters (OEB, CEB, SJ, SBJ, and FJT) of U14 and U17 female players remained unchanged regardless of the Ramadan period (Table 2). By conducting the ANOVA time X group analysis (Table 4), significant increases in PSQI, ISI, and ESS scores over time were observed for the U14 and U17 groups, with large effect sizes. Similarly, there was a significant decrease in sleep duration, corresponding to significantly later bedtimes over time, with large effect sizes. However, no changes were observed in the various physical parameters across the different periods.

## 4. Discussion

This is the first study to investigate the impact of the Ramadan fasting period on sleep patterns (PSQI, ISI, and ESS) in groups of adolescent girls under the age of 14 (U14) and under the age of 17 (U17). For both U14 and U17 players, results showed that sleep quality decreased, reflecting a significant increase in PSQI score during R1 (*p* < 0.01) and R4 (*p* < 0.05) compared to before Ramadan (BR). As expected, sleep habits undergo changes during the month of Ramadan, resulting in a reduction in the number of hours of sleep, with later bed and wake times [4,35,36,37,38]. With our female handball players, the results showed that bedtime became increasingly later than before Ramadan, while wake-up times remained strictly early due to school requirements that obliged our players to wake up too early to go to school. This led to a decrease in the number of hours of sleep during the R1 (*p* < 0.05) and R4 (*p* < 0.01) phases, contributing to an increase in the PSQI score for each age group studied. These results can be attributed to the prevalent dietary patterns in our Tunisian milieu, marked by a substantial upsurge (*p* < 0.05) in carbohydrate and sugar intake during R1 and R4, demonstrating a significant correlation with PSQI and ISI scores in U14 and U17 players (Table 3). These practices, encompassing dietary imbalances or excessive nutritional intake, may exert adverse effects on sleep patterns, as exemplified by [39].

More specifically, a high consumption of carbohydrates and sugar can lead to significant fluctuations in blood glucose and insulin, especially in young athletes aged U14 and U17. These glycemic fluctuations can disrupt the hormonal balance, particularly in terms of insulin and cortisol secretion. Elevated insulin levels can promote weight gain and increase the risk of obesity, which, in turn, can disrupt circadian rhythms and sleep cycles [40]. Furthermore, a sugar-rich diet can increase the production of free fatty acids in the blood, which can lead to systemic inflammation. This inflammation can affect the immune and nervous systems, thereby contributing to sleep-related issues. These findings are consistent with previous research indicating that dietary factors, such as the glycemic index of foods consumed, can significantly impact sleep quality and overall health [41]. Additionally, our findings align with a study conducted by [42], which indicated that fasting practices during Ramadan led to sleep alterations, including delayed sleep onset and reduced total sleep time among young athletes. This resonates with our observations in the Tunisian context.

In our study, a significant increase in ESS score was observed in U14 and U17 players during R1 compared to BR. However, during the last week of Ramadan (R4), this significant increase was observed only among U14 players, suggesting that the effects of fasting on sleepiness may vary with age or other factors over the course of Ramadan. These findings align with the existing literature, which consistently reports a correlation between inadequate sleep and increased daytime sleepiness, as well as decreased cognitive performance among adolescents. For instance, studies by [43,44] highlight that sleep deprivation significantly impacts diurnal alertness and cognitive function in younger populations. Additionally, research by [37,45,46] supports the notion that inadequate sleep can exacerbate daytime somnolence and impair cognitive abilities, particularly in adolescents facing altered sleep patterns due to external factors like fasting or academic demands. These studies, along with further research [47], emphasize that proper 24 h sleep hygiene practices can effectively mitigate these negative effects, underscoring the crucial role of maintaining good sleep habits throughout the entire day. This phenomenon can be elucidated by the substantial reduction in sleep duration, which progressively diminishes during Ramadan, thereby amplifying the requirement for sleep, particularly in individuals below the age of 14. Notably, the cumulative effect of losing 1 to 2 h of sleep daily can culminate in the manifestation of excessive daytime sleepiness (ESS > 8) by the conclusion of the Ramadan period [48]. Furthermore, apart from the data pertaining to our U17 players during R4, our findings diverge from previous studies, as they do not provide evidence of heightened daytime sleepiness independent of the Ramadan period, even in the presence of reduced sleep duration [36,49,50,51]. Another objective of our study was to determine the effect of Ramadan fasting on certain physical qualities among female players under 14 and under 17 years of age.

Regarding the quality of balance, our results showed that no difference was observed in the performances of the one-legged balance test with eyes open (OEB) and closed (CEB) during R1 and R4 compared to BR. This result is consistent with the study by [52], who used the same test protocol and found that balance performance was not affected during Ramadan in female athletes, despite the changes in diet and hydration status. However, this result is divergent from some previous studies [53,54] showing that postural control can be negatively affected during Ramadan in male judokas. The literature provides evidence related to abrupt changes in the circadian rhythm caused by late sleep, which occur mainly in adults and can impair anaerobic performance, including postural balance, in the case of partial sleep deprivation [21,55,56]. In our study, sleep duration significantly decreased during R1 and R4 for both groups compared to the BR period, and this decrease is related to changes in bedtime [35,36,51,57], becoming later in our female players as Ramadan progressed. We observed an average change of about one hour compared to the BR period while keeping the same wake-up times for all players in both groups. However, most previous studies involving adult subjects [35,36,49,51] have shown a delay of up to approximately 3 h in sleep during Ramadan compared to the BR period, which corresponds to a delay in wake-up times, which may explain the negative effect observed on some anaerobic measures, including postural balance. Hence, we postulate that the scholastic demands imposed on our athletes, mandating consistent wake-up schedules, exert a favorable influence on the preservation of anaerobic performance, encompassing the quality of postural balance. These consistent schedules and related sleep habits can be effectively monitored using wearable devices, providing valuable data on sleep patterns and their impact on athletic performance [58]. Other likely explanations for our results are related to psychological factors such as vigilance [4] and attention [59], which are known to be affected by fasting. As for the assessment of lower limb explosive strength, our findings indicate that jump performances (SJ, SBJ, and FJT) remained unaffected by the Ramadan period in both the U14 and U17 groups. These findings are consistent with those of other studies [12,13,20,60] showing that partial sleep deprivation has no impact on single-effort jump performances (SJ and CMJ). However, our results contradict the study conducted by Meckel et al. [1] on adolescents, which revealed a deterioration in jump performances based on CMJ during Ramadan.

The most cogent rationale underlying our results is associated with specific sleep parameters, including duration, bedtime, and wake-up time, which typify our players and potentially contribute to the preservation of jump performances, as has been previously established in the context of single-leg balance quality. The maintenance of physical performance observed in our players during Ramadan fasting may be attributed to several factors. Firstly, the players adhered to a consistent training schedule, with coaches prioritizing low- to moderate-intensity activities and minimizing prolonged, high-intensity, particularly aerobic, training sessions, especially during the hottest periods of the day. This training approach may have contributed to the preservation of physical capacities, particularly anaerobic performance. Secondly, the extended preparatory period of approximately 2 months prior to the start of the competitive season, which commenced about 7 months before Ramadan, likely played a significant role. This preparatory phase incorporated physical training focused on athletic activities such as plyometrics, which may have positively influenced the maintenance of physical performance during the Ramadan period. Considering that our players undergo a minimum of 12 h of food deprivation between their last meal and the commencement of training, one might have anticipated a notable decline in anaerobic performance due to potential factors such as dehydration and the depletion of glycogen reserves.

### 4.1. Strengths and Limitations of the Study

#### 4.1.1. Strengths

The results suggest that maintaining a regular low-intensity training program and limiting intense sessions are essential for preserving the physical performance of adolescent athletes throughout Ramadan. Coaches should ensure that athletes, particularly those in the U14 and U17 age groups, follow structured sleep schedules that include at least 8 h of sleep per night and sufficient rest. This is especially important due to the increased intake of energy, sugar, and glucose during this month.

#### 4.1.2. Limitations of the Study

Our study exhibits certain limitations that warrant consideration. Firstly, it is important to note that the period between BR, R1, and R4 is relatively long, approximately 3 months. Consequently, it is likely that the activity that occurred during this period may have an impact on the physical measurements taken between BR, R1, and R4. Moreover, the dietary data may be subject to reliability issues as they were collected over a limited timeframe. Additionally, we lack a comprehensive investigation into certain cognitive parameters related to Ramadan, such as the athletes’ attitudes and beliefs toward physical exertion. Furthermore, our study lacks comprehensive information regarding the control of sleep habits, including napping. To gain a more thorough understanding of the impact of Ramadan on fitness, future research should employ objective measurements of sleep parameters and incorporate assessments of psychological factors. Additionally, longitudinal studies could provide more detailed insights into how repeated fasting periods over the years affect the development and performance of adolescent athletes. It is also important to explore different age categories, including adults, to better understand the broader implications of Ramadan fasting on various life stages and athletic performance.

## 5. Conclusions

These findings suggest that maintaining a sleep duration of approximately 8 to 8.5 h during Ramadan, coupled with an early bedtime before midnight and regular waking hours compared to the normal baseline (BR), can assist young athletes in preserving their physical performance levels during Ramadan, particularly for anaerobic lactic efforts lasting less than 10 s. It has been proposed that the consumption of carbohydrate-rich foods during the evening meal for breaking the fast could aid in sustaining brief and intense physical performance at an optimal level. Carbohydrates serve as the primary energy source for intense physical activities, and dietary restrictions during Ramadan may affect the availability of these crucial nutrients for the body.

## Figures and Tables

**Table 1:** Anthropometric and dietary parameters before and during the first and last week of Ramadan in U14 and U17 groups.

Variables	U14			U17		
	BR	R1	R4	BR	R1	R4
Height (m)	1.64 ± 0.02	1.64 ± 0.04	1.64 ± 0.04	1.67 ± 0.07	1.67 ± 0.05	1.67 ± 0.05
Body mass (kg)	56.98 ± 4.90	57.41 ± 4.22	58.31 ± 4.21 **	59.55 ± 5.81	60.09 ± 5.25	62.09 ± 4.54 *
Body mass index (kg·m^−2^)	21.19 ± 1.42	21.42 ± 1.23	21.52 ± 0.81 **	21.71 ± 1.53	21.71 ± 0.96	21.99 ± 1.21 *
Body fat (%)	24.39 ± 1.51	24.49 ± 2.62	24.79 ± 3.11 **	22.18 ± 1.29	22.28 ± 2.50	23.90 ± 1.89 **
Lean body mass (kg)	43.90 ± 2.72	43.89 ± 2.85	43.09 ± 2.22	46.08 ± 3.18	46.89 ± 2.73	46.20 ± 2.89
Energy intake (kcal)	2652 ± 85	2802 ± 94	3100 ± 58	2792 ± 93	2995 ± 101	3102 ± 89
Protein (g)	115.02 ± 8.02	125.06 ± 9.01	127.02 ± 8.01 *	107.05 ± 11.01	99.00 ± 6.02	102.06 ± 9.04
Carbohydrates (g)	352 ± 25	399 ± 28 *	406 ± 31 *	375 ± 27	409 ± 33 *	422 ± 38 *
Sugar (g)	85 ± 18	121 ± 21 *	131 ± 23 *	89 ± 20	133 ± 25 *	135 ± 27 *
Lipids (g)	98.08 ± 8.07	100.03 ± 7.09	88.02 ± 7.01	93.04 ± 8.99	92 ± 11	108 ± 10

Significant differences from pre-Ramadan (BR) to the first week of Ramadan (R1) and last week of Ramadan (R4) are denoted as (*) *p* < 0.05 and (**) *p* < 0.01.

**Table 2:** Sleep and physical fitness before and during the first and last weeks of Ramadan in U14 and U17 groups.

Variables	U14			U17		
	BR	R1	R4	BR	R1	R4
PSQI (a.u.)	5.41 ± 1.48	5.89 ± 1.61 **	5.79 ± 1.58 *	5.37 ± 1.50	5.91 ± 1.52 **	5.70 ± 1.59 *
ISI (a.u.)	7.11 ± 2.59	7.32 ± 2.58 *	7.28 ± 2.08 *	7.51 ± 2.40	7.71 ± 2.31 *	7.69 ± 2.21 *
ESS (a.u.)	6.32 ± 1.90	6.86 ± 1.91 **	6.80 ± 1.72 *	6.32 ± 1.80	6.47 ± 1.50 *	6.30 ± 1.80
Sleep duration (h)	9.61 ± 0.42	8.88 ± 0.52 *	8.13 ± 0.72 **	9.01 ± 0.73	8.20 ± 0.59 *	7.42 ± 0.47 **
Bedtime (24-h)	21.92 ± 0.73	22.69 ± 0.82 *	23.51 ± 0.63 **	22.64 ± 0.53	23.32 ± 0.59 *	23.55 ± 0.78 **
Wake up time (24-h)	7.27 ± 0.49	7.39 ± 0.52	7.33 ± 0.40	7.43 ± 0.50	7.41 ± 0.42	7.28 ± 0.41
OEB (s)	10.88 ± 7.38	10.54 ± 6.64	10.56 ± 5.60	10.27 ± 7.44	9.51 ± 7.08	10.02 ± 5.52
CEB (s)	3.05 ± 1.02	3.81 ± 0.89	3.55 ± 1.52	2.29 ± 1.38	3.20 ± 0.86	3.18 ± 1.03
SJ (cm)	24.46 ± 4.80	23.53 ± 2.64	23.90 ± 3.23	28.11 ± 3.50	27.13 ± 2.88	27.40 ± 2.66
SBJ (m)	1.56 ± 0.18	1.54 ± 0.08	1.54 ± 0.22	1.66 ± 1.27	1.63 ± 0.12	1.64 ± 0.09
FJT (m)	9.18 ± 0.55	9.08 ± 0.84	9.17 ± 0.68	8.82 ± 0.89	8.82 ± 0.59	8.70 ± 0.82

Significant differences from pre-Ramadan (BR) to the first week of Ramadan (R1) and last week of Ramadan (R4) are denoted as (*) *p* < 0.05 and (**) *p* < 0.01. PSQI: Pittsburgh Sleep Quality Index; ISI: Insomnia Severity Index; ESS: Epworth Sleepiness Scale; OEB: open-eye balance; CEB: closed-eye balance; SJ: squat jump; SBJ: squat broad jump; FJT: five-jump test.

**Table 3:** Correlation matrix between sleep and nutrition parameter scores before and during the first and last weeks of Ramadan among U14 and U17 groups.

Group	Sleep	Period	Kcal	Protein	Carbohydrates	Sugar	Lipids
U14	PSQI	BR	0.231	0.010	0.132	0.097	0.047
		R1	0.580 **	0.054	0.421 *	0.513 **	0.197
		R4	0.612 **	0.192	0.580 **	0.455 *	0.063
	ISI	BR	0.333	0.120	0.292	0.128	0.093
		R1	0.411 *	0.202	0.351 *	0.391 *	0.106
		R4	0.384 *	0.144	0. 420 *	0.401 *	0.212
	ESS	BR	0.122	0.039	0.225	0.121	0.0152
		R1	0.234	0.092	0.312	0.081	0.228
		R4	0.232	0.082	0.283	0.105	0.125
U17	PSQI	BR	0.038	0.154	0.063	0.013	0.128
		R1	0.402 *	0.080	0.574 **	0.422 *	0.192
		R4	0.432 *	0.393	0.425 *	0.351 *	0.176
	ISI	BR	0.090	0.211	0.138	0.201	0.062
		R1	0.394 *	0.301	0.614 **	0.512 *	0.127
		R4	0.422 *	0.126	0.504 *	0.431 *	0.225
	ESS	BR	0.035	0.144	0.112	0.218	0.054
		R1	0.094	0.123	0.124	0.125	0.191
		R4	0.174	0.137	0.093	0.222	0.214

PSQI: Pittsburgh Sleep Quality Index; ISI: Insomnia Severity Index; ESS: Epworth Sleepiness Scale; kcal: average energy intake over 24-h; BR: before Ramadan; R1: first week of Ramadan; R4: last week of Ramadan. The correlations are significant at the 0.05 (*) or 0.01 (**) levels (two-tailed).

**Table 4:** Sleep and physical fitness in U14 and U17 groups according to time effect.

Variables	U14				U17			
	ANOVA × Time				ANOVA × Time			
	R1		R4		R1		R4	
	[F (*p*)]	η2	[F (*p*)]	η2	[F (*p*)]	η2	[F (*p*)]	η2
PSQI (a.u.)	13.052 (0.008) **	0.152	12.068 (0.014) *	0.142	15.582 (0.006) **	0.162	12.528 (0.035) *	0.147
ISI (a.u.)	11.122 (0.038) *	0.139	11.989 (0.037) *	0.142	10.052 (0.045) *	0.141	11.588 (0.024) *	0.152
ESS (a.u.)	17.542 (0.007) **	0.164	13.458 (0.021) *	0.150	17.022 (0.042) *	0.159	9.988 (0.062)	0.100
Sleep duration (h)	21.722 (0.04) *	0.138	23.252 (0.007) **	0.157	24.248(0.01) *	0.139	26.152 (0.004) **	0.155
Bedtime (24-h)	15.676 (0.03) *	0.162	17.176 (0.005) **	0.185	14.026 (0.03) *	0.147	16.156 (0.002) **	0.182
Wake up time (24-h)	7.952 (0.09)	0.082	6.292 (0.06)	0.102	9.022 (0.07)	0.092	10.596 (0.06)	0.120
OEB (s)	9.392 (0.07)	0.085	10.158 (0.09)	0.082	8.692 (0.06)	0.091	10.088 (0.06)	0.101
CEB (s)	11.055 (0.07)	0.096	11.158 (0.09)	0.102	10.033 (0.08)	0.082	12.028 (0.06)	0.110
SJ (cm)	8.982 (0.59)	0.124	10.552 (0.07)	0.109	11.782 (0.09)	0.098	10.958 (0.06)	0.121
SBJ (m)	7.254 (0.09)	0.110	8.607 (0.09)	0.114	10.021 (0.07)	0.109	7.998 (0.059)	0.121
FJT (m)	10.462 (0.09)	0.106	13.528 (0.06)	0.110	13.762 (0.08)	0.082	11.652 (0.07)	0.108

Significant differences from pre-Ramadan (BR) to the first week of Ramadan (R1) and last week of Ramadan (R4) are denoted as (*) *p* < 0.05 and (**) *p* < 0.01. PSQI: Pittsburgh Sleep Quality Index; ISI: Insomnia Severity Index; ESS: Epworth Sleepiness Scale; OEB: open-eye balance; CEB: closed-eye balance; SJ: squat jump; SBJ: squat broad jump; FJT: five-jump test.

## Data Availability

The data that support the findings of this study are available from the corresponding author upon reasonable request.
